# No evidence of antigenic seniority in hemagglutinin specific antibody responses after adjuvanted pandemic 2009 influenza vaccination

**DOI:** 10.1016/j.jvacx.2019.100029

**Published:** 2019-06-20

**Authors:** Anders Madsen, Linda Azimi, Sarah Tete, Fan Zhou, Florian Krammer, Rebecca Jane Cox, Åsne Jul-Larsen

**Affiliations:** a Influenza Centre, Department of Clinical Science, University of Bergen, Norway; b K.G. Jebsen Centre for Influenza Vaccine Research, Department of Clinical Science, University of Bergen, Norway; c Department of Microbiology, Icahn School of Medicine at Mount Sinai, New York, NY, USA; d Department of Research and Development, Haukeland University Hospital, Bergen, Norway

**Keywords:** Healthcare worker, Antigenic seniority, AS03, Pandemic vaccine

## Abstract

Influenza is a contagious respiratory illness caused by the influenza virus. The pandemic outbreak of influenza A H1N1 in 2009 (H1N1pdm09) gave us a unique opportunity to study humoral immune responses to a novel influenza vaccine strain. Here, we investigate how an individual’s previous encounter with different influenza subtypes influences the humoral response after pandemic vaccination in 2009.

We retrospectively chose and grouped 80 vaccinated healthcare workers (HCWs) based on their year of birth into 4 groups, reflecting which influenza subtype they were likely first exposed to during childhood. Pre- and 21 days post- vaccination sera were analyzed. We investigated antibodies to the major surface protein hemagglutinin (HA), and specifically antibodies binding to the conserved stalk domain of the HA-protein. Serological assays were used to assess the quantity and functionality of the influenza-specific antibodies, including virus neutralization and activation of natural killer (NK) cells involved in antibody-dependent cell-mediated cytotoxicity (ADCC).

The AS03-adjuvanted H1N1pdm09 vaccine elicited robust antibody responses in all groups of HCWs. We found that the more antigenically experienced individuals had higher pre-vaccination antibody-levels towards the stalk domain of the HA. We also demonstrated that despite their inferior pre-vaccination antibody levels, the younger individuals reached similar antibody levels as the older birth-cohorts after pandemic vaccination. Our findings are important for understanding the effect of AS03 adjuvant on the antibody response in individuals exposed to different influenza viruses during their early childhood years, which is crucial for developing vaccine strategies against influenza.

## Highlights

• AS03-adjuvanted H1N1pdm09 vaccine elicited robust antibody responses.
• More antigenically experienced individuals had higher pre-vaccination HA stalk specific antibodies.
• AS03 adjuvanted H1N1pdm09 vaccine overcomes problems of antigenic seniority.

## 1 Introduction

Influenza is a contagious respiratory disease causing annual epidemics with an estimated 300,000–600,000 deaths each year [1]. However, morbidity and mortality can increase dramatically when, at an unpredictable interval, a pandemic occurs. In April 2009 a novel influenza A H1N1 virus emerged (H1N1pdm09), with high transmissibility among humans. The virus spread globally, and a pandemic was declared on June 11th 2009 [2]. Vaccination against influenza can reduce infection, disease severity and death, and remains the main method of prophylaxis against infection by inducing B cell responses leading to the production of neutralizing antibodies. In 2009, Norway initiated a mass vaccination campaign to protect the population. Around 2.2 million people were vaccinated with the AS03 adjuvanted monovalent pandemic influenza vaccine during the pandemic, and healthcare workers (HCW) were prioritised for receiving the first round of vaccination before the peak of pandemic virus activity [3].

Antibodies targeting the conserved stalk domain of the major influenza surface protein hemagglutinin (HA) can provide broad protection against diverse influenza A subtypes. This may be important during pandemic outbreaks when novel viruses emerge [4], [5], [6], [7]. The antibody response to seasonal trivalent influenza vaccination (TIV) is mainly focused to the receptor-binding site on the head domain of HA, which limits vaccine efficacy to the virus strains included in the vaccine [8]. The HA head specific antibodies inhibit infection by neutralizing the virus, and are traditionally measured by the hemagglutination inhibition (HI) assay [9]. The HA stalk specific antibodies can provide protection by blocking viral fusion with the host cell and by eliminating the infected host cells through antibody-dependent cellular cytotoxicity (ADCC) [10].

An individual’s immune defense against influenza is also shaped by their previous infections with different influenza viruses during their lifetime. The circulating influenza subtype during childhood years (priming strain) may play an important role in protection against novel influenza A viruses later in life. The term “original antigenic sin” has been used to describe how infections later in life can give an elevated response to the childhood priming strain, instead of solely eliciting a response to the newly encountered strain [11]. More recently, the term “antigenic seniority” has been used to describe the phenomenon of how the priming strain has a more “senior” role in the adaptive immunity to subsequent infections [12]. The clinical importance of the childhood priming strain is clearly illustrated by studies measuring how birth-year predicts the risk of severe illness or death against novel influenza viruses [13], [14].

There is, however, limited data on how an individual’s previous antigenic experience affects different aspects of the HA-specific antibody response to pandemic vaccination. In this study we vaccinated HCWs with the AS03 adjuvanted monovalent pandemic influenza vaccine and investigated the HA-specific antibody response in relation to their previous antigenic experience reflected by their birth-year.

## 2 Material and methods

### 2.1 Study population

Eighty participants were selected from a clinical trial where HCWs were vaccinated with the AS03 adjuvanted monovalent pandemic split H1N1 virus (A/California/7/2009-like virus, X179a) vaccine (Pandemrix, GlaxoSmithKline (GSK), Belgium) at Haukeland University Hospital, Bergen, Norway. The inclusion and exclusion criteria have been previously published [15]. Subjects who had virologically confirmed H1N1pdm09 were not prioritised for vaccination due to the limited vaccine availability during 2009 and were excluded from the study. All HCWs provided written informed consent before inclusion in the study. The study was approved by the regional ethics committee (Regional Committee for Medical Research Ethics, Western Norway (REK Vest 2012/1772) and the Norwegian Medicines Agency. The trial is registered in the European Clinical Trials Database (2009-016456-43), and National Institute for Health Database Clinical trials.gov (NCT01003288). Serum samples were collected pre- and 21 days post- vaccination and stored frozen until used in this study.

### 2.2 HA proteins and influenza viruses

Chimeric cH9/1 protein (trimeric HA protein composed of a H1 stalk domain from A/Puerto Rico/8/1934 (H1N1) and a H9 head domain from A/guinea fowl/Hong Kong/WF10/99) and a H1 HA (trimeric A/California/04/09) were generated using the baculovirus expression system [5], [7]. Recombinant baculoviruses were passaged three times through Sf9 cells, before infection of High Five cells. Purified proteins were analyzed using sodium dodecyl sulfate–polyacrylamide gel electrophoresis (SDS-PAGE) and quantified by infrared spectrometer (DirectDetect®, Milipore Corporation).

Recombinant cH9/1N3 (virus neutralization assay and ADCC reporter assay), and A/California/07/2009(H1N1) (hemagglutination inhibition and microneutralization assays) viruses were propagated in embryonated hen’s eggs 10 days after fertilization.

### 2.3 HI assay

Antibodies targeting the receptor binding site of HA, detected in the hemagglutination inhibition (HI) assay were tested against the H1N1pdm09 strain (inactivated A/California/7/2009) using 2-fold serial dilutions of receptor destroying enzyme (Seiken, Japan) treated sera, and 0.7% turkey red blood cells, as previously described [15]. The HI-titer was defined as the reciprocal of the highest dilution of serum to prevent 50% agglutination. Negative HI-titers (<10) were assigned a value of 5 for calculation purposes.

### 2.4 Endpoint ELISA

The hemagglutinin specific serum IgG titer was measured by indirect enzyme-linked immunosorbent assay (ELISA), as previously described [10]. The 96-well plates (Nunc maxisorp™) were coated with 1 μg/ml of HA protein and incubated overnight before adding 5-fold serial dilutions of HCW sera. Horseradish peroxidase (HRP) conjugated monoclonal mouse anti human IgG (BD Pharmingen™) were added to the HA reactive antibodies and detected by adding a colorimetric substrate (3,3′,5,5′-3,3′,5,5′-tetramethylbenzidine (TMB)) (BD Biosciences, USA). The endpoint-titer was defined as the reciprocal of the highest dilution of serum to give a detectable measurement (an optical density (OD) > 3 standard deviations above the mean of blank controls).

### 2.5 Avidity ELISA

The avidity of the hemagglutinin specific IgG was measured by avidity ELISA [16]. Sera were standardised to an OD of 0.7 ± 0.3 using an indirect ELISA. After a one hour incubation, sera were treated with 1.5 M chaotropic agent NaSCN (Sigma, St Louis, MO, USA). The avidity Index was calculated as the percentage of antibody remaining bound after treatment; (OD_Treated serum_/OD_Untreated serum_) × 100%.

### 2.6 Microneutralization

The microneutralization (MN) assay was performed using H1N1pdm09 like-virus (X179a) as previously described [17]. Sera were heat inactivated at 56 °C for 30 min and added in 2-fold serial dilutions to a 96-well plate (Nunc maxisorp™) with virus (2000TCID_50_/ml). Madin-Darby Canine Kidney (MDCK) cells were added after one hour, and plates were further incubated for 16–18 h. Cells were fixed with hydrogen peroxide the next day, and infected cells were detected by the presence of influenza A nucleoprotein, mouse anti-influenza A nucleoprotein antibodies were incubated with the fixed cells for one hour at 37 °C before adding HRP-labeled secondary antibody, and detected by adding TMB. MN-titers were calculated as the dilution of serum at which 50% of MDCK cells were infected. Negative MN-titers (MN-titer < 10) were assigned a value of 5 for calculation purposes.

### 2.7 cH9/1N3 virus neutralization

The cH9/1N3 virus neutralization (VN) assay was performed as previously described [10]. In short, 1000 TCID_50_/ml of cH9/1 N3 virus was incubated with 2-fold serial dilutions of heat-inactivated sera in viral growth medium (Dulbeco Dulbecco’s Modified Eagle’s Medium with tosyl phenylalanyl chloromethyl ketone-trypsin, 0.14% bovine serum albumin, 100 units/ml penicillin, 100 μg/ml streptomycin and 0.25 μg/ml amphotericin B) for one hour at 37 °C. The serum-virus mixture was added to a 96-well plate with confluent MDCK cells and incubated for one hour at 37 °C. The virus and sera were then washed away before adding new medium with the same serum dilution to the MDCK-cells and incubating for 72 h. Then, 50 µl of the supernatant was transferred to a 96-well V bottom plate to measure the hemagglutination activity. The VN-titer was defined as the highest dilution of serum causing 100% hemagglutination (using 0.7% human 0- blood).

### 2.8 ADCC NK cell activation assay

ADCC assay for intracellular natural killer (NK) cell interferon-gamma (IFNγ) and CD107a expression was conducted as previously described, and analyzed with the same gating strategy [10]. Briefly, 96-well plates were coated overnight at 4 °C with chimeric cH9/1 HA protein (1 μg/ml). The next day, plates were washed with PBS and incubated with heat-inactivated sera (sera dilution 1:10) for two hours at 37 °C. Plates were washed again with PBS and incubated with 10^5^ CD16 176v NK-92 cells per well (mycoplasma-free, human NK cell line expressing high affinity 176 V variant CD16 receptor) (Fox Chase Cancer Center, Philadelphia, PA, USA). As a negative control, NK-92 cells lacking expression of CD16 were added to an additional well for each sample.

The cells were incubated with anti-CD107a-AF488 antibody (Biolegend, San Diego, CA, USA), Brefeldin A (5 μg/ml, BD) and monensin (5 μg/ml, BD) for 16 h at 37 °C. After incubation, cells were stained with LIVE/DEAD Fixable Aqua dead cell staining kit (Invitrogen), anti-CD3-PE CF594 (BD) and anti-CD56-APC (BD) before intracellular staining with anti-IFN-γ-BV-421 (Biolegend). Cells were acquired on BD Fortessa. Data analysis was done using FlowJo version 10 (treeStar).

### 2.9 ADCC reporter assay

The ADCC reporter assay was performed as previously described, with minor modifications [18], [19]. The assay measures luciferase activity as a reporter for activation of the nuclear factor of activated T-cells (NFAT) pathway following Fc-receptor (FcγRIIIa) binding of antigen specific antibodies. In short, MDCK cells were seeded into 96-well white flat-bottom plates and incubated at 37 °C. After 18–24 h, cells were infected with cH9/1N3 virus at a multiplicity of infection (MOI) of one for 30 min. On the next day, serial dilutions of sera in ADCC Assay Buffer (Roswell Park Memorial Institute (RPMI) 1640 Medium with 4% Low lgG fetal bovine serum (FBS)) was added to the plate and incubated at 37 °C for 30 min. ADCC Bioassay effector cells (Jurkat) resuspended in ADCC Assay Buffer were added to the assay plate. After incubation at 37 °C for 6 h, Bio-Glo™ Luciferase Assay Reagent (Promega) was added, and luminescence (RLU) was read. A curve of the RLU versus antibody dilution factors was made in order to determine the EC_50_ of the antibody response.

### 2.10 Statistical analysis

Statistical analyses were performed using Graphpad Prism version 6 for Mac. For each group of healthcare workers, the D0 to D21 response to vaccination was assessed using paired nonparametric Wilcoxon test. Differences between groups were tested with one-way ANOVA using Tukey test for multiple comparisons. P < 0.05 was considered statistically significant.

## 3 Results

### 3.1 Demographics of the healthcare workers

**Fig. 1:**
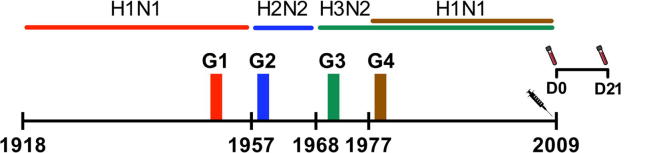
Study outline. Four groups of healthcare workers (Group 1; G1, Group 2; G2, Group 3; G3 and Group 4; G4) were retrospectively selected according to their year of birth (G1; 1947–1952, G2; 1958–1960, G3; 1969–1972 and G4; 1978–1980), in relation to circulating influenza subtypes. The dominating influenza A subtypes circulating from 1918 and prior to 2009 are shown. All healthcare workers (n = 20 in each group) received the AS03 adjuvanted monovalent pandemic influenza vaccine in 2009. Serum samples were collected before vaccination (D0) and 21 days (D21) after vaccination.

Eighty HCWs were divided into four groups (n = 20) based on their year of birth, reflecting which influenza A subtype they were likely to be immunologically primed with during early childhood (Fig. 1). As the highest attack rates are reported in young children [20], we grouped subjects so that they were at least 5 years old at the start of the next pandemic. The HCWs in this study were mostly female (78%), except for group 3, which had an even gender distribution (Table 1). This reflects the gender distribution of Norwegian healthcare workers in general. The majority (66%) of the subjects received seasonal influenza vaccine prior to 2009. Four individuals (5%) were vaccinated with the 2009 seasonal vaccine, as well as the pandemic vaccine. Two individuals (2.5%) reported underlying medical conditions.

**Table 1:** Demographics of study participants.

Characteristic	Group 1 (n = 20)	Group 2 (n = 20)	Group 3 (n = 20)	Group 4 (n = 20)
Female/Male (ratio)	18/2 (9.0)	17/3 (5.7)	10/10 (1.0)	18/2 (9.0)
Year of birth	1947–1952	1958–1960	1969–1972	1978–1980
Number of major Influenza A subtypes experienced	4	3	2	2
Mean age vaccinated pandemic vaccine (year range)	60.3 (57–62)	49.7 (49–51)	38.0 (37–39)	30.0 (29–31)
Previous seasonal influenza vaccination before 2009 (yes/no/unknown)	15/5/0	17/3/0	11/8/1	11/9/0
Seasonal vaccination in 2009 (yes/no)	0/20	0/20	2/18	2/18
Underlying medical conditions* (yes/no)	1/19	1/19	0/20	0/20
Working in infectious disease department (yes/no)	1/19	0/20	3/17	2/18

* One subject in group 1 reported respiratory disease, and one subject in group 2 reported immunological disease.

### 3.2 AS03 adjuvanted pandemic vaccine elicited a potent antibody response in all groups of HCWs

**Fig. 2:**
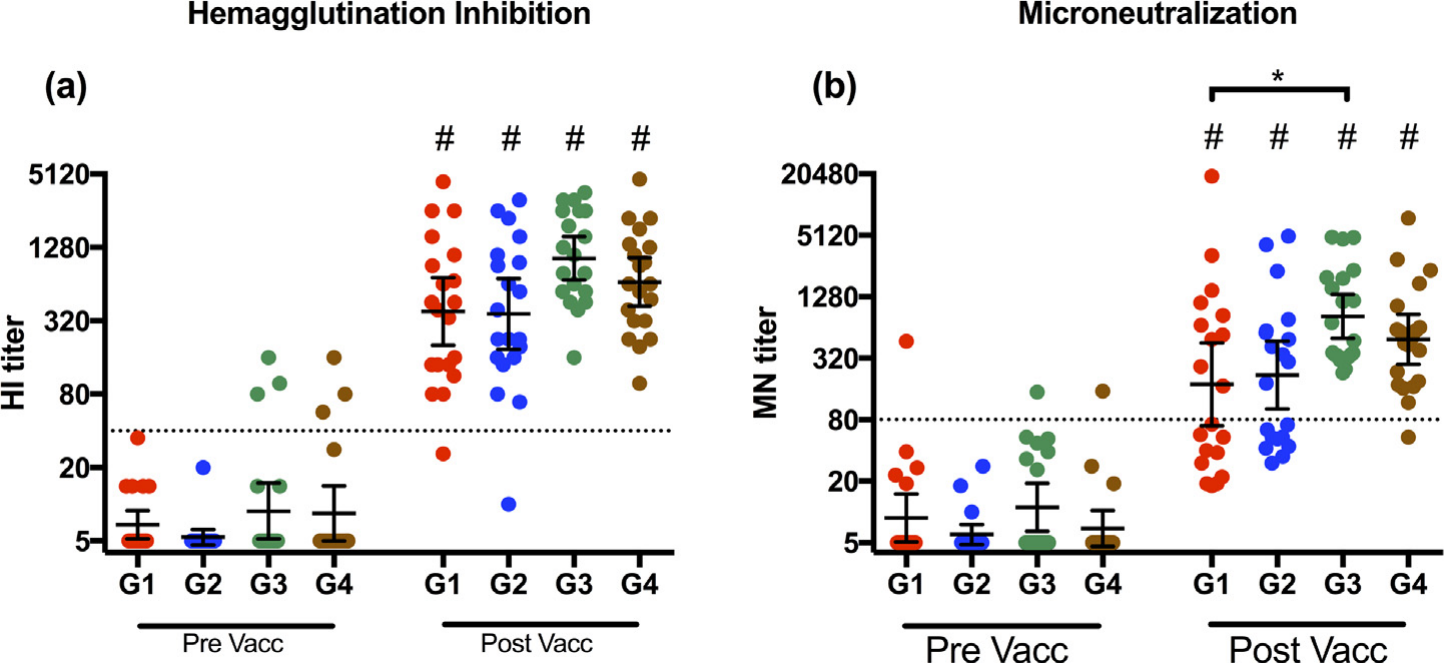
Haemagglutination inhibition and Microneutralization response after vaccination. Haemagglutination Inhibition (HI) titers (A) and microneutralization titers (B) against A/California/7/09 (H1N1 pdm09 virus) after pandemic influenza vaccination. Four groups of healthcare workers (Group 1; G1, Group 2; G2, Group 3; G3 and Group 4; G4) were retrospectively selected according to their year of birth (G1; 1947–1952, G2; 1958–1960, G3; 1969–1972 and G4; 1978–1980). Serum samples were collected pre- and 21  days post vaccination. Each data point represents a single individual. The dotted lines represents surrogate correlates of protection (HI titer of 40 and MN titer of 80). The geometric mean titre (GMT) ± 95% confidence intervals are shown. ^*^, *p <* 0.05. # Indicates statistical significance (p < 0.05) from pre to post vaccination.

We examined the H1N1pdm09- specific HI response after AS03 adjuvanted pandemic vaccination (Fig. 2a). Prior to vaccination, 81% of the HCWs had no detectable antibodies to the H1N1pdm09 virus. HI titers of ≥40 are deemed a protective titer in adults with a 50% reduction in the risk of influenza in a human challenge study [21]. Only 8% of the HCWs had an HI titer above the protective level of 40. No significant differences in the pre-vaccination HI titers were observed between the groups. Vaccination resulted in a significant increase (p < 0.0001) in the HI titers in all 4 groups of HCWs. The geometric mean titer (GMT) of the HI titers were highest in groups 3 (1040) and 4 (666), and similar in groups 1 (382) and 2 (364). The HI-titers in all 4 groups met all three criteria of the committee for Medical Products for Human Use (CHMP) for immunogenicity of pandemic vaccines [22], which are seroconversion rate >40%, seroprotection rate >70% and a geometric mean ratio >2.5 from pre- to post-vaccination (Supplementary Table 1).

The microneutralization assay was performed to determine the functionality of the H1N1pdm09 specific antibodies (Fig. 2b). The majority (78%) of the HCWs had no detectable pre-vaccination neutralizing antibodies (MN-titer < 10) with only 3 individuals (4%) having MN-titer ≥80, which has been suggested to be protective [23]. Vaccination resulted in a significant increase in MN titers in all groups (p < 0.0001). The highest GMTs were found in groups 3 and 4 (GMT = 826 and 491, respectively). Furthermore, group 3 had significantly higher GMT than group 1 (p < 0.05). The percentage of subjects with an MN-titer ≥80 after vaccination was lower in groups 1 and 2 (50% and 55%, respectively) compared to groups 3 and 4 (100% and 95%, respectively). The H1N1pdm09 specific MN titers correlated significantly with the HI titers; Spearman’s R = 0.735, P < 0.0001 pre-vaccination, and Spearman’s R = 0.924, P < 0.0001 for post vaccination (data not shown).

### 3.3 All groups elicited a robust HA-specific IgG antibody response, despite pre-vaccination differences

**Fig. 3:**
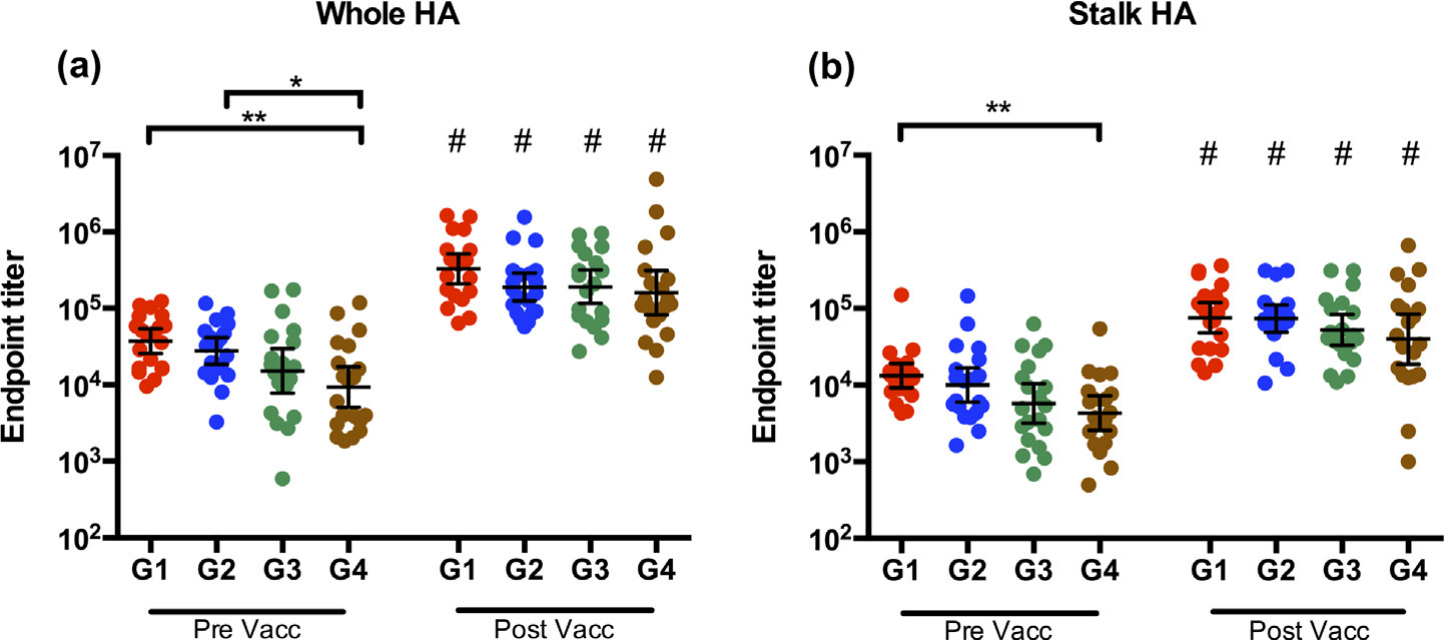
Influenza HA-specific IgG concentrations after vaccination. The concentration of serum IgG specific to (a) the whole HA of H1N1pdm09 and (b) the stalk HA of H1N1pdm09 measured by ELISA. Four groups of healthcare workers (Group 1; G1, Group 2; G2, Group 3; G3 and Group 4; G4) were retrospectively selected according to their year of birth (G1; 1947–1952, G2; 1958–1960, G3; 1969–1972 and G4; 1978–1980). Serum samples were collected pre- and 21 days post vaccination. Each data point represents a single individual. The geometric mean titre (GMT) ± 95% confidence intervals are shown. ^*^, *p <* 0.05; ^**^, *p* < 0.01. # Indicates statistical significance (p < 0.05) from pre to post vaccination.

We investigated the influenza HA-specific serum IgG antibodies in the HCWs after H1N1pdm09 vaccination (Fig. 3) by measuring the antibody response against the whole HA (Fig. 3a) and the HA stalk using the chimeric HA (cH9/1) (Fig. 3b). A gradual decreasing trend in pre-vaccination antibody titers from group 1 to group 4 was observed. Groups 1 and 2 had significantly higher pre-vaccination antibody titers against whole HA compared to group 4, p < 0.01 and p < 0.05, respectively. The pre-vaccination titers were also significantly higher in group 1 than group 4 against the HA stalk (p < 0.05). Vaccination significantly increased (p < 0.0001) the HA whole and stalk specific antibodies in all groups. After pandemic vaccination, no significant difference in the titers of HA specific IgG was found between the groups.

### 3.4 Vaccination increased the quality and functionality of the HA-stalk specific antibodies

**Fig. 4:**
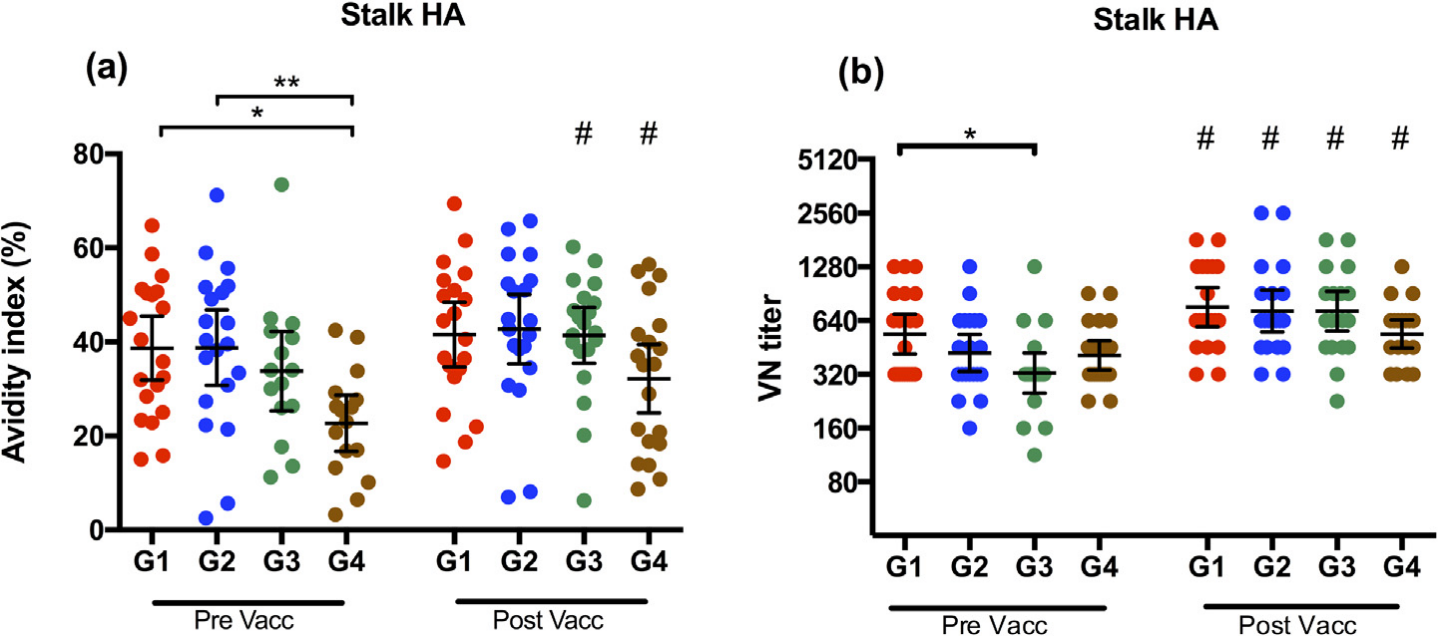
HA-stalk specific IgG avidity and ability to neutralize virus after vaccination. (A) The avidity of influenza specific IgG to the HA stalk of H1N1pdm09 determined by NaSCN-elution ELISA. The avidity index is the proportion of serum antibodies remaining bound after treatment with chaotropic agent NaSCN. (B) Virus neutralization titres against cH9/1 N3 virus. Four groups of healthcare workers (Group 1; G1, Group 2; G2, Group 3; G3 and Group 4; G4) were retrospectively selected according to their year of birth (G1; 1947–1952, G2; 1958–1960, G3; 1969–1972 and G4; 1978–1980). Serum samples were collected pre- and 21 days post vaccination. Each data point represents a single individual. The geometric mean titre (GMT) ± 95% confidence intervals are shown. ^*^, *p <* 0.05; ^**^, *p* < 0.01. # Indicates statistical significance (p < 0.05) from pre to post vaccination.

We analyzed the HA-stalk-specific IgG antibodies by measuring the antibody-antigen binding-strength (avidity) in ELISA (Fig. 4a). The antibody avidity index was measured as the proportion of serum antibodies remaining bound to HA after treatment with a denaturizing agent (1.5 M NaSCN). Prior to vaccination, groups 1 and 2 had the highest avidity index, with a mean of 38.7% and 38.8% respectively, which was significantly higher than group 4 (mean avidity index = 22.7%). Vaccination significantly enhanced the antibody avidity in HCWs of groups 3 and 4 (p < 0.05), but not groups 1 and 2. Group 4 had the lowest antibody avidity index 21 days after vaccination (32.2%), although not significantly different from the other groups.

The VN assay was performed using a chimeric virus (cH9/1N3) and a prolonged incubation time (72 h), in order to detect the functional stalk specific antibodies capable of preventing conformational changes of the HA, inhibition of viral egress and hindering cleavage activation of the HA [4]. With this high-sensitivity assay we were able to detect neutralizing stalk antibodies pre- vaccination in all subjects (Fig. 4b). Group 1 had the highest pre-vaccination VN-titers, which were significantly higher (p < 0.05) than group 3. Vaccination resulted in a significant increase in all groups of HCWs (p < 0.01). Group 4 had the lowest post vaccination titers, although no significant differences between the groups were found.

### 3.5 H1N1pdm09 vaccine induced antibodies involved in NK cell activation

**Fig. 5:**
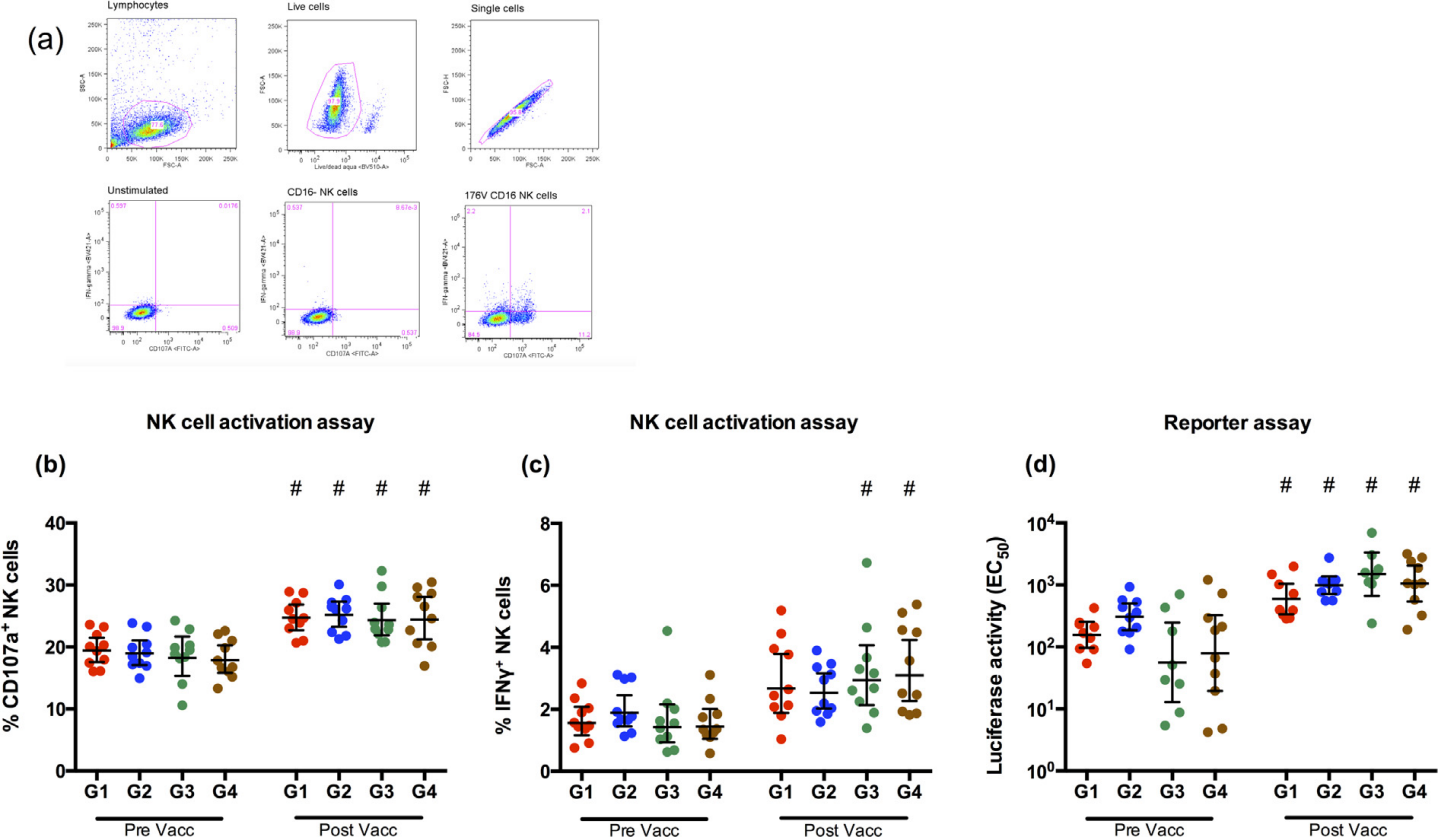
Indirect neutralization by HA stalk specific antibodies though ADCC. Serum antibodies ability to induce HA stalk specific ADCC, measured as frequencies of NK cells expressing (B) CD107a or (C) IFNγ with NK cell activation assay using cH9/1 protein, and (D) by measuring luciferase activity with reporter assay (promega kit) using cH9/1 N3 virus. Four groups of healthcare workers (Group 1; G1, Group 2; G2, Group 3; G3 and Group 4; G4) were retrospectively selected according to their year of birth (G1; 1947–1952, G2; 1958–1960, G3; 1969–1972 and G4; 1978–1980). Serum from samples were collected from the four groups of healthcare workers pre- and 21 days post vaccination. Each data point represents a single individual. The geometric mean titre (GMT) ± 95% confidence intervals are shown. # Indicates statistical significance (p < 0.05) from pre to post vaccination.

We further dissected the functionality of the HA stalk specific antibodies by measuring the ADCC inducing antibodies, as a possible mechanism of protection. Firstly, we performed an NK activation assay on 10 randomly chosen subjects from each group by coating the plates with cH9/1 HA and measuring the CD107a (Fig. 5b) and IFNγ (Fig. 5c) expression. The gating strategy is shown in Fig. 5a. We observed no pre-vaccination differences between the groups. There was a significant boost of CD107a expression post-vaccination in all groups (p < 0.05). There was also an increase in IFNγ expression after vaccination, but only significant pre to post vaccination changes were observed in groups 3 and 4.

Secondly, we performed the high-sensitivity ADCC reporter assay on the same 10 individuals, measuring antibodies ability to activate the NFAT pathway (Fig. 5d) using a recombinant cH9/1N3 virus. With this assay we were able to see differences pre-vaccination. Groups 1 and 2 had the highest levels of luciferase activity, although not significant compared to groups 3 and 4, which had more individual differences. All groups showed a significant increase in activity after vaccination.

## 4 Discussion

In this study, we have investigated how priming to different circulating influenza subtypes during childhood influences the immune response to adjuvanted pandemic H1N1 vaccination in HCWs. We vaccinated HCWs prior to the peak of pandemic activity with the AS03 adjuvanted pandemic H1N1pdm09 vaccines, and grouped HCWs that were born 5 or more years prior to a new pandemic outbreak during the 20th century, as Bodewes et al. found highest attack rates of influenza A viruses in children between 2 and 3 years old [20].

HCWs are an important risk group with high occupational exposure to influenza that were prioritised for the first rounds of vaccination against pandemic influenza [24].

We found that 15% of the HCWs in groups 3 and 4 had protective HI-titers to H1N1 pdm09 prior to vaccination. This is possibly due to cross-protection from previous vaccines, or due to subclinical infection through occupational exposure as patients with suspected H1N1pdm09 infection were admitted to the hospital 10 weeks before pandemic vaccination started [25]. Of note, no individuals in groups 1 and 2 had HI-titers above the protective threshold prior to pandemic vaccination. There was larger proportion of healthcare workers working in infectious disease ward from groups 3 and 4 (12.5%) compared to groups 1 and 2 (2.5%), although none of these individuals had protective HI-titers before vaccination (Table 1).

The AS03 adjuvanted H1N1pdm09 vaccine elicited high HI and MN titers in all groups. The AS03 functions by effectively activating the innate immune system, increasing antigen uptake and presentation by monocytes in the local draining lymph nodes [26]. There are two mechanisms in which the AS03 adjuvant particularly elicited a good humoral response; firstly by stimulating increased activation of naïve B cells and thereby overcoming the problems of previous influenza immunity. Secondly, the use of an adjuvant increases the adaptability of recalled memory B cells leading to the fine-tuning of the lineage specificity through further rounds of affinity maturation [27]. As a result, H1N1pdm09 vaccine elicited B and CD4 T cell responses that were higher than non-adjuvanted H1N1pdm vaccines, and cross-reacted with previous H1N1 strains dating back to 1977 [28], [29].

To better understand the effect of AS03 adjuvanted H1N1pdm09 vaccine on antigenic seniority, we investigated IgG antibodies targeting the conserved HA stalk domain of the H1N1pdm09 virus. In ELISA we found a gradual decrease in GMT with decreasing age, from groups 1 to 4 pre-vaccination. These results are consistent with other studies investigating the effect of age and total antigenic experience [6], [30]. However we did not observe the same trend as clearly in the neutralization assays (Fig. 2). The discrepancy is probably due to the different aspects of antibody levels that the experiments measure. The neutralization assay measures the functionality of the antibody response, whereas the ELISA-assay measures quantity. Despite these pre-vaccination differences, all groups reached similar levels of HA stalk and whole HA-specific IgG 21 days after vaccination with AS03 adjuvanted vaccine. This suggests that the antibody levels reached a ceiling after vaccination, which has been previously observed [31].

Antibody binding strength may be an important indicator of protection against disease. High antibody avidity has been associated with increased virus neutralization and milder disease upon infection during the 2009 influenza pandemic [32]. When examining the avidity to the HA stalk of H1N1pdm09, we found a significantly higher pre-vaccination avidity (p < 0.05) in groups 1 and 2, compared to group 4. This suggests that they were better primed for this antigen [33].

We further dissected the HA stalk antibodies by examining their ability to neutralize virus and activate NK cells, which are important for prevention and clearance of infections. Other birth-year cohort studies have found a significant increase of cross-reactive antibodies against H1N1pdm09 with age, which were highest in older adults who were probably previously exposed to a 1918-like H1N1 virus [34], [35].

In our pre-vaccination samples we could see a trend of increased antibody functionality, both neutralization and ADCC activity, with antigenic experience (Fig. 5C).

Understanding the impact of antigenic seniority on the antibody response to a novel adjuvanted influenza vaccine is important for the development of better vaccine strategies. The older individuals who had likely been exposed to more diverse HAs had superior quantity and quality of the HA stalk specific antibodies before pandemic vaccination than the youngest group. We also demonstrate that the AS03-adjuvanted vaccination was beneficial in all groups of HCWs, and led to a significant increase in the HA specific antibodies.

In our study, the individuals born before emergence of H3N2 in 1968 were primed with HA (H1N1 or H2N2) that belonged to the same phylogenetic group as the H1N1pdm09 virus (group 1 HA). This could explain the higher HA stalk-neutralization pre-vaccination titers compared to individuals of Group 3, as stalk reactive antibodies have been found to cross-react with other influenza subtypes within the same phylogenetic group [5]. Neutralizing stalk antibodies can rise modestly over time and be boosted through immunization with HA that have shared stalk domains and different head domains [5], [6].

The population size (n = 80) of 20 per group allowed differences to be analyzed, although a larger study is needed to confirm these results. We have made the assumption that all study subjects were infected with influenza within 5 years after they were born. However, it is possible that some individuals remained immunologically naïve to influenza for more than 5 years after birth, and therefore got primed with a different influenza subtype than our grouping would suggest (Fig. 1). Immunosenescence can be a confounding factor when measuring the effect of antigenic experience, as Immunosenescence can affect humoral immunity in older individuals [36]. Although, whether this is relevant for individuals in Group 1 (aged 57–62), is not clear [37]. Age differences could explain the elevated post-vaccination HI, MN and ADCC levels in groups 3 and 4 compared to groups 1 and 2. Klein et al. reported that the humoral response to vaccination is higher in women compared to men [38], although this was not reflected in findings for group 3 which had an equal male to female distribution.

AS03, as a potent adjuvant, elicited a robust antibody response regardless of age and birth year grouping. Further studies with non-adjuvanted H1N1pdm09 vaccines are needed before we can conclude how the AS03 adjuvant has impacted the responsiveness with regards to antigenic seniority. Moreover, the 21-day time-point is a short interval after vaccination. Therefore, investigation of long-term antibody responses are needed to better understand the impact of antigenic seniority. It would also be helpful to include more cellular immunity in future studies, as this plays a vital role in disease severity. We have investigated the impact of antigenic seniority on vaccine immunogenicity at the individual antibody level. Further investigation is needed to confirm our findings at the population level, and how it affects disease severity and death. A strength of this study is that we assessed the humoral response using five immunological assays, covering both the quantity, quality and functionality of the antibodies.

In summary, our findings provide insight to how an individual’s potential previous encounter with different influenza subtypes shapes the cross-reactive humoral immunity to a novel influenza virus and influences the response to pandemic vaccination. This can be important for future public health strategies during a pandemic.
