# Supplementary material for: No evidence of antigenic seniority in hemagglutinin specific antibody responses after adjuvanted pandemic 2009 influenza vaccination
Source: Vaccine X. 2019 Jun 20;2:100029. doi: 10.1016/j.jvacx.2019.100029 (PMC6668305; doi:10.1016/j.jvacx.2019.100029)
Supplement: Supplementary Data 1 [file mmc1.docx]

**Supplementary table 1: CHMP criteria fulfillments**
